# Efficacy of a single dose versus a multiple dose regimen of Mebendazole against hookworm infections among school children: a randomized open-label trial

**DOI:** 10.1186/s12879-020-05097-1

**Published:** 2020-05-27

**Authors:** Tegegne Eshetu, Mulugeta Aemero, Ayalew Jejaw Zeleke

**Affiliations:** Department of Medical Parasitology, School of Biomedical and Laboratory Sciences, College of Medicine and Health Sciences, University of Gondar, P.O.Box: 196, Gondar, Ethiopia

**Keywords:** Hookworm, Single dose, Multiple dose, Mebendazole, Efficacy

## Abstract

**Background:**

Despite the existence of a population-based control program using single dose albendazole or mebendazole as a preventive chemotherapy, hookworm transmission remains high. It causes a negative impact on the growth and school performance of children. In connection to this preventive chemotherapy, different studies produced conflicting results. This study aimed at evaluating the efficacy of single (500 mg) versus multiple doses (100 mg twice a day during three consecutive days) of mebendazole against hookworm infections among school-aged children.

**Methods:**

This randomized open-label clinical trial took place among school-aged children (6–14 years old) in Burie and Debre Elias towns, Northwest Ethiopia. Using simple randomization, eligible hookworm-positive children were allocated (1:1) to either a single or multiple dose treatment arms. Stool samples were collected and processed using McMaster method at baseline and follow-up period (14–21 days after treatment). Only laboratory technicians were blinded. The cure and egg reduction rates were the primary and secondary therapeutic outcome measures against hookworm infections, respectively. An independent t-test was used to compare group means, and logistic regression was used to calculate odds ratio (OR). *P*-value < 0.05 at 95% CI was considered statistically significant.

**Result:**

One hundred eight children, 54 in each treatment arm had completed baseline data and received allocated treatment. One hundred three children had completed follow-up data records and included for the final efficacy analysis. Cure rate against hookworm was significantly higher in the multiple dose (96.1%) than in the single dose (30.8%) with OR = 55.125; 95% CI: 11.92–254.9; *P* < 0.001. The egg reduction rate in the multiple dose treatment arm (99.5%) was also significantly higher than in the single dose arm (68.9%) with difference t (101) =5.38; 95% CI 230.95–505.36; *P* < 0.001.

**Conclusion:**

The single dose regimen of mebendazole for the treatment of hookworm infections showed poor cure and egg reduction rates, while the multiple doses revealed satisfactory. Although multiple dose regimen administration is a bit more complex than the single dose, we strongly encourage replacing it with multiple dose regimen during deworming programs in hookworm endemic areas.

**Trial registration:**

This trial is retrospectively registered in www.pactr.org, number PACTR201911466695052 on November 26, 2019.

## Background

Globally, hookworm’s disease burden remains high, and around 500 million people are infected [1]. In 2017, the global burden of hookworm infections was estimated at 845,000 disable-adjusted life years (DALYs) [2]. This parasite is mainly associated with hypo-albuminemia, iron deficiency anemia, and malnutrition, which cause more subtle chronic health problems like physical and intellectual growth retardation in children, and adverse pregnancy outcomes [3, 4]. Moreover, it causes annual productivity loses with estimated range of $7.5 billion to $138.9 billion [1]. The highest burden and intensity of infections occur in sub-Saharan Africa followed by Asia, Latin America and the Caribbean [5]. In sub-Saharan Africa, it has been estimated that 40–50 million school-aged children and 7 million pregnant women are infected [6]. In general, over 267 million preschool-aged children and over 568 million school-aged children live in areas where the parasite is intensively transmitted and are in need of treatment and preventive interventions [7].

Currently, control efforts for hookworm infections are implemented through periodic mass drug administration using either of a single dose of albendazole (400 mg) or mebendazole (500 mg) regimens [8–10]. This control measure is implemented without prior information on the infection status of individuals. These days, different studies have revealed conflicting results on the impact of preventive chemotherapy (PC) [4].

In addition to this, multiple studies have shown that, although cheap and widely available, the single dose of mebendazole is unsatisfactorily effective against hookworm infections, with CRs ranging from 18 to 59% [10–15].

A multiple dose (100 mg twice a day over three consecutive days) of mebendazole is among the recommended and widely used anthelminthic regimen for treating hookworm and other soil-transmitted helminthes (STHs) infections throughout the world [16, 17]. Only a limited numbers of studies were conducted to evaluate the multiple dose efficacies against hookworm infections. The available literature reveals CR ranging from 26 to 97.9% and ERR from 85 to 100% [14, 18, 19]. This inconsistent efficacy status of the drug warrants further studies.

Previously, only one randomized clinical trial comparing the efficacy of the single dose to the multiple dose of mebendazole against hookworm has been conducted [18]. However, it is the only randomized clinical trial conducted so far. To contribute to an increase of evidence, we also conducted a clinical trial comparing the single to the multiple dose of mebendazole against hookworm. This study is the first of its kind in Ethiopia.

## Methods

### Study design

This randomized, open-label clinical trial was conducted at Burie and Debre Elias towns’ primary schools, Northwest Ethiopia, from March to May, 2019. This study included school-aged children aged 6 to 14 years. The study was approved by the Ethical and Review Committee of School of Biomedical and Laboratory Sciences, College of Medicine and Health Sciences, University of Gondar, Ethiopia. This trial is retrospectively registered in www.pactr.org, number PACTR201911466695052 on November 26, 2019.

Prior to participant enrolment, all parents/legal guardians’ of the children were informed about the objective, purpose, study procedures, and the potential risk and benefits of participating in the study. Parents/legal guardians who agreed that their child should be enrolled in the study were asked to sign a written informed consent. Verbal assent was also sought from each participant. Parents/legal guardians who were unable to read and write, were asked to give thumbprint after having been read the full informed consent form by a data collector.

### Intervention, trial medication, and outcome measures

This randomized clinical trial had two treatment arms: (i) a single dose of mebendazole (500 mg) and (ii) a multiple dose of mebendazole (100 mg twice a day for three consecutive days). The multiple dose of mebendazole (WORMIN tab) was commercially obtained from a private pharmacy in the local market, while the single dose of mebendazole (Vermox®) was provided by the local coordinator office of the deworming program. The primary outcome considered was CR against hookworm, while ERR for determining the changes in infection intensity served as a secondary outcome measure 14–21 days of post treatment.

### Eligibility criteria and sample size

Eligible for inclusion were all hookworm-positive children with a signed informed consent who did not have additional health problems (based on medical history, physical examination, vital signs). The following exclusion criteria were also applied: children who received any form of anthelminthic treatment within the past 30 days, were unable to chew the drug (for the single), had diarrhea at the time of the first sampling, had a hemoglobin level < 8 g/dl, experienced a severe concurrent medical condition or had any known history of allergic reaction to mebendazole, and infected with other parasitic infection. All remaining children were randomized and allocated to one of the two treatment arms.

The desired sample size was determined by using WHO guidelines [20]. The local prevalence of hookworm infections was not exactly known, and hence, it was assumed to be 50%. Fifty from each of the two treatment arms would be enough to detect differences in the CR with 80% power using a 2-sided statistical test with alpha-level of 0.05. Moreover, considering a potential loss to follow-up, 20% as a non-response rate was added. Finally, 300 school-aged children were screened for hookworm infections to get the minimum required sample size.

### Data collection and laboratory procedures

Study participants responded to a short questionnaire investigating demographic and other health-related issues using the WHO drug efficacy assessment from. A unique identification number was given to each participant. Then, each participant received a sterile stool container labeled with his/her unique identification number and was asked to provide approximately 10 mg of fresh stool. All children were well informed on how to avoid any contamination of the sample. Samples were immediately transported to the nearby health center laboratory in Debre Elias and Burie hospital laboratory.

The McMaster concentration technique, which is the standard reference method for evaluating drug efficacy in Veterinary Parasitology and has recently been evaluated for human helminthes, was used for this study [20, 21]. Laboratory quality control was performed by an expert microscopist by re-reading 10% of slides of each laboratory technician. Only one stool sample was collected from each participant at baseline and follow-up.

Upon completion of all the baseline parasitological and participant information survey, hookworm-positive children were subjected to a physical and clinical examination by a senior health officer. Height was measured with a standard meter (to the nearest 0.1 cm), and weight with an electronic balance (to the nearest 0.1 kg). Haemoglobin levels were measured in capillary blood using the finger-prick method (HemoCue®301). Children who were found positive for *Ascaris lumbericoide* and *Trichuris trichiura* were treated with albendazole (400 mg) during baseline screening.

### Randomization

Using simple randomization lottery techniques, eligible hookworm-positive children were randomly assigned either to the single (500 mg) or multiple dose regimen of mebendazole (100 mg twice a day for three consecutive days) arm with a 1:1 ratio. Eligible children were randomly assigned and allocated to each treatment arm by the researcher. The drug administrator and children were not masked for drug treatment. Only laboratory technicians were blinded to the dose allocation, hypothesis and objective of the study.

### Drug administration

A slice of biscuit was given to each eligible child before drug administration. The single dose and the first dose of the multiple dose of mebendazole regimens were administered to each randomized child in front of their parents by the research team and public health officer at school. After administering the drug, children in the single dose were monitored for 3 to 4 h to observe if any vomiting and other adverse events were occurred following treatment. In the case of children who were randomized into multiple dose arm; parents/guardians were convinced to take home the remaining tablets in a sealed envelope and were instructed on how to administer the drugs. They were instructed to give the drug twice a day (every morning and evening for 3 days), avoid skipping/doubling any dose, refrain from giving any kind of alcohol, and follow strictly their child up to the end of treatment. Participants/parents/guardians were informed to report any medical discomfort following treatment to the investigators or the nearby health extension worker.

### Follow-up data collection

Each treated child was revisited 14–21 days after drug administration and asked to provide one stool sample for the second time. At this time point, children were also asked about the occurrence of vomiting and diarrhea following drug administration.

A participant who vomited within 4 h after drug administration or a participant with diarrhea was excluded for the final analysis. The same laboratory procedures took place at the follow-up. Children who remained infected with hookworm or other STH were treated with albendazole (400 mg) at the end of the study.

### Statistical analysis

Data was entered to Epi-data software to check data completeness and clearance, and thentransferred to SPSS version-23 for statistical analysis. All analyses were performed on a per-protocol basis. Only children with complete data sets were included in the analysis to determine the treatment efficacy. The baseline characteristics of the study participants are summarized using frequencies, mean and standard deviation (Table 1). Infection intensity with hookworm was grouped in to light, moderate and heavy infections, according to WHO guidelines [22]. Cure and egg reduction rates were used to assess the efficacy of the drug. Cure rate was assumed to be the proportion of individual hosts positive for hookworm who become parasitologically negative after treatment [23]. Egg reduction rate was defined as the relative reduction in the mean egg output after treatment compared to pre-treatment value [24]. ERR was expressed using both the arithmetic mean (AM) and the geometric mean (GM) [25].

**Table 1:** Baseline characteristics of randomized children, at Burie and Debre Elias towns, North West Ethiopia, January–June 2019

	Single dose (*N* = 52)	Multiple dose (*N* = 51)
Sex		
Male	17 (32.7%)	28 (54.9%)
Female	35 (67.3%)	23 (45.1%)
Mean (SD) age, year	10.78 (2.1)	10.48 (1.34)
Mean (SD) weigh, kg	31.45 (7.96)	29.69 (6.2)
Mean (SD) height, m	1.35 (0.13)	1.34 (0.08)
Mean (SD) haemoglobin, g/dl	13.8 (1.09)	14.02 (0.93)
Baseline EPG		
Arithmetic mean (95% CI)	1216.35 (845.84–1586.86)	1134.3 (864.4–1404.2)
Geometric mean (95% CI)	826.1 (651.5–1039.7)	821.3 (656.3–1024.8)
Infection intensity		
Light (1–1999 EPG)	43 (82.7%)	45 (88.2%)
Moderate (2000–3999)	7 (13.5%)	5 (9.8%)
Heavy (> = 4000)	2 (3.8%)	1 (2%)

*SD* Standard deviation.

Confidence intervals for ERR were calculated using bootstrap re-sampling method with 5000 iterations.

An independent t-test was used to compare group means, whereas CRs were compared by calculated Odds Ratio (OR) using logistic regression. For all statistical analyses a *P-value* of 0.05 was considered as the limit for statistical significance.

## Results

**Fig. 1:**
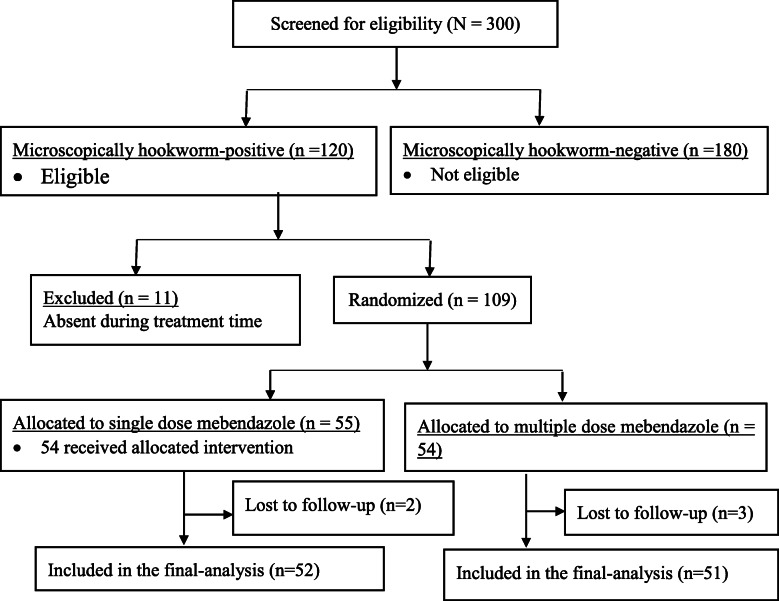
Flow chart for identification procedures of eligible children

A total of 300 school-aged children were enrolled in the baseline screening. Of these, 120 (40%) (64 females and 56 males) were found to be hookworm-positive. Eleven hookworm infected children were excluded because they were absent from school on the clinical and physical examination day. From 109 randomized children, one child from the single dose of mebendazole arm was not willing to receive the allocated treatment. Thus, 108 eligible children, 54 in each treatment arm had completed baseline data and received allocated treatment. Fourteen to twenty one days of post treatment, follow up sample collection was performed with a 7 day time frame. During this time, two participants in the single dose and one child in the multiple dose arms were absent from school and two children in the multiple dose arm were unable to provide sufficient stool samples. Finally, a total of 103 children had complete data records, 52 in single dose and 51 in multiple dose mebendazole arms were included for the final efficacy analysis (Fig. 1).

### Baseline survey

At baseline, the eligible children allocated in the two treatment groups were comparable in terms of several characteristics. The mean age in single dose group was 10.78 ± 2.1 years and 10.48 ± 1.34 years in the multiple dose group. The mean weights of participants for single and multiple doses were 31.45 ± 7.96 and 29.69 ± 6.2 kg, respectively. Moreover, most participants were diagnosed with light hookworm infections (see Table 1).

### Efficacy of multiple and single dose regimen of mebendazole for treating hookworm infections

The follow-up fecal egg intensity of hookworm in terms of arithmetic mean were 378.04 EPG and 5.88 EPG in the single and multiple dose arms, respectively. Cure rates of the single and multiple dose regimens of mebendazole for treating hookworm infection were 30.8% (19.2–44.2%) and 96.1% (90.2–100%), respectively. In addition, the ERRs in arithmetic mean were 68.9% in the single dose and 99.5% in the multiple dose arms. The ERR in geometric mean were Overall, 36 (69.2%) school-aged children who were treated with single dose and two (3.9%) treated with multiple dose mebendazole remained hookworm-egg positive at follow-up (see Table 2).

**Table 2:** Cure and egg reduction rates of single and multiple dose mebendazole against hookworm infections among school-aged children from Burie and Debre Elias towns, January – June 2019

	Single dose	Multiple dose
No. of Infections before treatment (%)	52 (100%)	51
No. of cured after treatment	16	49
CR (95% CI)	30.8 (19.2–44.2)	96.1 (90.2–100)
EPG arithmetic mean		
Before treatment (95% CI)	1216 (845.8–1586.86)	1134.3 (864.4–1404.2)
After treatment (95% CI)	378.04 (237.1–510.99)	5.88 (− 2.85–14.6
ERR(95% CI)	68.9% (48.07–73.14)	99.5% (98.97–100)
EPG geometric mean		
Before treatment (95% CI)	826.1 (651.1–1039.7)	821.3 (656.3–1024.8)
After treatment (95% CI)	61.3 (27.8–133.0)	0.4 (0.0–1.0)
ERR (95% CI)	92.6% (87.5–96.4)	99.9% (99.8–100)
Infection intensity after treatment	N (%)	N (%)
Light (1–1999 EPG)	35 (67.1%)	3 (5.9%)
Moderate (2000–3999)	1 (1.9%)	–
Heavy (> = 4000)	–	–

There was a substantial difference for both CR and ERR between multiple and single dose regimen of mebendazole for treating hookworm infections [(CR: 96.1% versus. 30.8%; OR = 55.125; 95% CI: 11.92–254.9; *p* < 0.001), (ERR = 99.5% versus 68.9%; 95% CI 230.95–505.36; *p* < 0.001)].

## Discussion

Ethiopia is a hotspot area for hookworm and other STH infections. School children are disproportionately affected by these parasites [6, 26]. Mass drug administration for selected risk groups, such as children, using a single dose of albendazole or mebendazole is the mainstay for the control of STHs in Ethiopia [27]. However, there are recent reports which showed a reduction of the efficacy of the single dose mebendazole efficacy in some endemic areas [12, 14, 18, 28]. Moreover, an increased use of the single dose of mebendazole in many endemic areas may lead to the developments of drug resistance. Thus, perhaps other regimens of the drug could increase its efficacy. This calls for continuous monitoring of its therapeutic efficacy.

So far, few studies have investigated the efficacy of the multiple dose of mebendazole against hookworm infections [18, 29, 30]. The present study showed that a multiple dose of mebendazole (CR = 96.1%, CI: 90.2 to100) is significantly more efficacious at clearing hookworm infections than the single dose (CR = 30.8%, CI: 19.2–44.2) with OR = 55.125; 95% CI: 11.92–254.9; *P <* 0.001. Almost all hookworm-infected children were cured following multiple dose of mebendazole treatment. Our results are in line with the only head to head comparative randomized controlled trial study, which revealed superiority of the efficacy status of the multiple dose over the single dose mebendazole [18].

On the other hand, the therapeutic efficacy of the multiple dose in the current study is considerably higher than those previously reported in Iran(CR = 35% & ERR = 40.83%) [30] and Brazil (CR = 58.5%) [29]. This inconsistency in efficacy results could be related to the use of different diagnostic techniques, the sample size variation, the age of study participants, the parasite genetic diversity, and the study site. For instance, the study conducted in Iran applied the Stoll diagnostic technique, whereas the study in Brazil used the duplicated Kato-Katz and Hoffmann’s spontaneous sedimentation techniques. Thus, variation in the sensitivity of the diagnostic techniques might be the possible source for the discrepancies.

Studies have been conducted to assess the efficacy of the single dose of mebendazole on hookworm infections [14, 18, 19, 28, 31–33]. Overall, their CRs ranged from 7.6 to 70.3% and ERRs ranged from 52 to 76.3%. The CR of the single dose in our trial was 30.8% (CI 19.2 to 44.2%) and the ERR was 68.9% (CI 48.1 to 73.1%). This is in line with studies conducted in China (CR = 29%) [14], Vietnam (CR = 38% & ERR = 52%) [19], and Tanzania (CR = 24.4 & ERR = 59.5%) [32]. However, it does not agree with other studies conducted in Zanzibar (CR = 7.6%) [28], Tanzania (CR = 13%) [18], Lao PDR (CR = 17.6% & ERR = 76.3%) [33], and Cameroon (CR = 70.3%) [31]. The above mentioned reasons could also have resulted in the discrepancies.

Lower efficacy of the single dose compared to the multiple doses of mebendazole might be associated with the extensive and frequent use of the single dose in deworming program. Although administration of the single dose of mebendazole for mass treatment is convenient in terms of practical implementation, our findings reveal that its capacity to achieve its primary objective of preventive chemotherapy on intensity reduction is questionable. In other words, 43 (82.7%), of the infected study participants were under light infection category at the base line, and 35 (67.1%) remained in this category after treatment (Table 2).

The administration of multiple doses of mebendazole as mass chemotherapy seems to be complex and the cost of administration is likely to be higher. However, our results strongly encourage its use as a preventive and control measure in hookworm endemic areas. In addition, this tangible efficacy variation in our trial also indicating new option for administering a multiple dose of mebendazole regimen in the prevention and control programs through handing the drugs to teachers or parents as we did in this trial in order to minimize the cost and other logistic issues.

Although comparing the two commonly used dose of mebendazole in a head to head manner is considered as strength of our study, the infection intensity of the parasite was determined by the examination of a single stool sample. Also, the multiple dose of mebendazole was administered by caregiver of each participant and the tablets might be not administered as recommended. This might have affected our findings which should be interpreted with these limitations in mind.

Overall, the multiple dose regimen of mebendazole showed satisfactory efficacy with significantly higher CR and ERR than the single dose regimen, against hookworm infections in school-aged children. These results advocate a need to revise treatment guidelines of the current deworming programs, particularly in hookworm endemic areas. Moreover, we recommend conducting further studies using a larger sample size, more sensitive diagnostic procedures and in different regions of the globe.

## Data Availability

The data generated or analyzed during this study is included in this manuscript. Other data will be available from the corresponding author upon request.

## Abbreviations

AM: Arithmetic Mean
BZ: Benzimidazole
CI: Confidence Interval
CR: Cure Rate
DALYs: Disable Adjusted Life Years
EPG: Egg per Gram
GM: Geometric Mean
ERR: Egg Reduction Rate
OR: Odds Ratio
PC: Preventive Chemotherapy
STH: Soil-Transmitted Helminths
WHO: World Health Organization
